# Will the EU AI Act help to mitigate dataset bias in medical AI?

**DOI:** 10.1093/jlb/lsag001

**Published:** 2026-02-12

**Authors:** Emilia Niemiec, Peter A E Davis, Mathias K Hauglid

**Affiliations:** Centre for Advanced Studies in Bioscience Innovation Law (CeBIL), Faculty of Law, University of Copenhagen, Karen Blixens Plads 16, 2300 København S, Denmark; Centre for Advanced Studies in Bioscience Innovation Law (CeBIL), Faculty of Law, University of Copenhagen, Karen Blixens Plads 16, 2300 København S, Denmark; Wikborg Rein Advokatfirma AS, Dronning Mauds Gate 11, 0250 Oslo, Norway

**Keywords:** AI act, bias, data governance, debiasing exception, medical AI systems, sensitive data

## Abstract

The aim of this article is to provide an overview and analyze the implications of the provisions on dataset quality and bias in the AI Act (AIA). The AIA requires providers of AI systems to take measures to identify, prevent, and mitigate biases as part of the data governance practices. The AIA also explicitly prescribes certain characteristics required of training, validation, and testing datasets. These include notions widely considered as best practice such as representativeness as well as consideration of characteristics particular to the “geographical, contextual, behavioural or functional setting” which might expand the scope of considerations already common among AI developers. The AIA also aims to address the legal limitations on access to sensitive data by introducing the so called “debiasing exception,” which under certain conditions permits the processing of sensitive data for debiasing purposes. To ensure enforcement of the data governance provisions, the AIA grants notified bodies and enforcement authorities access to training, validation, and testing datasets; however, further efforts may be needed to reconcile data protection concerns with these enforcement powers. The AIA’s requirements will likely help mitigate bias in medical AI systems. Associated soft law instruments should contribute to the effective implementation of these requirements.

## I. INTRODUCTION

“Garbage in, garbage out” is a frequent adage in the artificial intelligence (AI) space, underscoring the fact that the performance of AI systems is contingent on the quality of the data they are trained on. Datasets used to develop medical AI systems, however, may not be of adequate quality in terms of representativeness of the intended patient population, labeling accuracy, missing information about the patients (such as about comorbidities or ethnicity if relevant^1^), or training datasets may not be independent from testing datasets.^2^ Insufficiently robust datasets used to train and validate an AI system may negatively impact the system’s performance and its generalizability to different subgroups.

Obtaining and implementing datasets of adequate quality poses challenges. Availability of existing datasets with desired characteristics may be limited, while collecting data prospectively is a laborious and costly process. Furthermore, questions arise about what patients’ characteristics should be considered to ensure that the data are representative. For example, should ethnicity be routinely reported in datasets used to build medical AI systems? Researchers have recently called upon regulators to provide guidelines on this issue^3^ and mandate testing the AI systems on different subgroups.^4^

To address the overlapping problems of data quality and discriminatory bias in medical AI systems, standards, guidelines, and initiatives have been developed. Standardization organizations have been working on documents addressing bias in AI systems, such as ISO/IEC 12791 and IEEE P7003. Reporting guidelines for various types of medical AI studies, such as MI-CLAIM,^5^ CONSORT-AI,^6^ and TRIPOD + AI^7^ also stress the importance of adequately describing the data used to develop and test an AI system. Recent guidelines on clinical evaluation by Collins et al. (2024) specifically address fairness of medical AI systems and indicate that differences in performance should be examined in major subgroups of the intended population, for example in sex and ethnic groups, which would reveal potential biases.^8^ There are also frameworks that specifically address data management.^9^

Not least of all, lawmakers have also taken measures to address the problem of data quality and bias in medical AI systems. While AI is often covered by regulation in legal areas such as data protection and privacy, consumer protection, and sector-specific law (including in the medical field),^10^ many jurisdictions have begun to enact omnibus AI-focused regulation to deal more directly with its many and emerging challenges. Dataset quality and bias are common focuses within these efforts, including the recently enacted Artificial Intelligence Act (AIA)^11^ in the European Union. The AIA explicitly addresses both dataset bias (Article 10) and automation bias (ie, “automatically relying or over-relying on the output produced by a high-risk AI system”) (Article 14(4)(b), AIA). Initial analysis of data quality requirements in the European Commission’s proposal for the AIA has been presented by van Kolfschooten.^12^ Van Bekkum has recently discussed the AIA’s debiasing exception, however, without addressing the enforcement considerations.^13^ In this article, we provide an overview and analyze the implications and problems raised by the provisions on dataset quality and dataset bias in the final text of the AIA. We present and analyze: (1) the AIA and its data governance and quality provisions, (2) the debiasing exception, and (3) the conformity assessment, post-market requirements, and enforcement considerations. This work’s main contribution to the research field is: (a) the examination of the final version of the AIA’s data governance provisions in the context of medical AI; (b) the analysis of the related enforcement provisions and identification of potentially contradictory requirements. As is well-documented, the EU stands as a regulatory superpower,^14^ and the AIA is set to influence global norms pertaining to AI. This analysis, therefore, has implications well beyond Europe.

## II. AIA AND DATA GOVERNANCE REQUIREMENTS

The AIA adds several new facets^15^ to an already comprehensive set of pre- and post-market requirements that apply to medical AI systems under the Medical Devices Regulation (MDR) and *In Vitro* Diagnostic Medical Devices Regulation (IVDR).^16^ Medical software that is within the scope of the MDR or IVDR and simultaneously meets the definition of “AI system” in the AIA must meet the requirements of both instruments.^17^ In the remainder of this article, we focus on such systems referred to as “medical AI systems.”

The AIA classifies AI systems according to their risk (Recital 26, AIA). In practice, most medical devices that leverage AI are likely to fall within the high-risk category, which entails the most detailed regulatory requirements. This is because the AIA establishes that AI systems falling within the scope of the MDR or IVDR and requiring a third-party conformity assessment (which applies to most currently available medical AI systems^18^) are to be classified as high-risk AI systems (Article 6(1), AIA). The AIA’s obligations for high-risk medical AI systems (including on data quality) will apply from 2 August 2027 (Article 113(c), AIA). There is, however, amnesty granted for such AI systems placed on the market before August 2 2026. If these systems are not intended for use by public authorities, they will not be subject to the requirements for high-risk systems of the AIA, unless they undergo significant changes in their designs after that date. If the systems are intended for use by public authorities (eg, public hospitals), their providers must implement the AIA's requirements for high-risk systems by 2 August 2030 (Article 111(2), AIA).

Under the MDR and IVDR, dataset quality and bias are addressed only implicitly, through the requirements for safety and performance of medical AI systems (Annex I, Chapter I, Section 1, MDR, and IVDR). These may not be met if biased or insufficient training, validation, or testing datasets are used to develop such systems. The AIA goes further by introducing provisions which explicitly address data quality and aim to mitigate dataset bias in the development of AI systems, including medical AI systems.

Dataset quality and bias are tackled most directly in Article 10 of the AIA, headed “Data and data governance.” This provision demands that providers (essentially, the system’s developers—see Article 3(3), AIA) implement “data governance and management practices appropriate for the intended purpose of the high-risk AI system” and specifies that these practices concern training, validation, and testing datasets (Article 10(2), AIA). The AIA defines these three types of data(sets) (Article 3(29–32), AIA; Table 1). This is pertinent as “validation” and “testing” have been given different meanings across various regulatory contexts and scientific domains.^19^ The AIA, however, does not define the term “bias,” which is also used in Article 10 (see below and Table 2).

**Table 1:** AI Act’s definitions of different types of data(sets)

**Data(set)**	**Definition**
Training data	“data used for training an AI system through fitting its learnable parameters” (Article 3(29))
Validation data	“data used for providing an evaluation of the trained AI system and for tuning its non-learnable parameters and its learning process in order, inter alia, to prevent underfitting or overfitting” (Article 3(30))
Validation dataset	“a separate data set or part of the training data set, either as a fixed or variable split” (Article 3(31))
Testing data	“data used for providing an independent evaluation of the AI system in order to confirm the expected performance of that system before its placing on the market or putting into service” (Article 3(32))

**Table 2:** Article 10(2) of the AIA

**Data governance and management practices shall concern^^a^^:**
“(a) the relevant design choices; (b) data collection processes and the origin of data, and in the case of personal data, the original purpose of the data collection; (c) relevant data-preparation processing operations, such as annotation, labeling, cleaning, updating, enrichment and aggregation; (d) the formulation of assumptions, in particular with respect to the information that the data are supposed to measure and represent; (e) an assessment of the availability, quantity and suitability of the data sets that are needed; (f) examination in view of possible biases that are likely to affect the health and safety of persons, have a negative impact on fundamental rights or lead to discrimination prohibited under Union law, especially where data outputs influence inputs for future operations; (g) appropriate measures to detect, prevent and mitigate possible biases identified according to point (f); (h) the identification of relevant data gaps or shortcomings that prevent compliance with this Regulation, and how those gaps and shortcomings can be addressed.” (Article 10(2))

^a^Exact wording of the beginning of Article 10(2): “Training, validation and testing data sets shall be subject to data governance and management practices appropriate for the intended purpose of the high-risk AI system. Those practices shall concern in particular…”

Article 10(2) includes a list of data management practices (Table 2), of which two points explicitly address bias. Point (f) requires providers to examine potential biases, outlining three types of potential biases that should be considered (Table 2). In the context of medical AI systems, biases “that are likely to affect the health and safety of persons” are most relevant of these three. An AI-driven diagnostic software that gives a substantially greater proportion of false negatives to individuals of a particular sex or ethnicity serves as one such illustration of this type of bias.

The subsequent point (g) further requires that, upon identifying biases under point (f), “appropriate measures to detect, prevent and mitigate” these biases should be part of the provider’s data governance and management practices. However, the provision refrains from specifying what those practices should involve, using only the term “appropriate,” which implies that the measures will depend on the specific context. Article 10(2)(g) also implies a recognition by the EU lawmaker that seeking to entirely eliminate the forms of bias specified in point (f) through legislative fiat is impossible.

While the data management practices in Article 10(2) are formulated rather vaguely, the subsequent paragraphs (3) and (4) of Article 10 explicitly prescribe certain characteristics required of training, validation, and testing datasets (Table 3). Adhering to these requirements, which include notions widely considered as best practice such as representativeness and lack of errors, will likely help minimize the risk of bias in datasets. Moreover, these data management practices required by the AIA might expand the scope of considerations already common among AI professionals. Particularly, Article 10(4) encourages the consideration of a wide range of aspects, including the “geographical, contextual, behavioural or functional setting” in which an AI system is intended to be used. Documenting the consideration of each of these aspects could prove to be a daunting task for developers, however, this provision could have positive effects reaching beyond the mitigation of biases in individual AI systems. It may force the stakeholders in medical AI to think carefully about where biases might come from and how they might impact the health and safety of patients in AI-driven healthcare.

**Table 3:** List of requirements for datasets included in Article 10(3) and (4)

**Training, validation, and testing datasets shall:**
be relevant (Article 10(3)) be sufficiently representative (Article 10(3)) be free of errors to the best extent possible (Article 10(3)) be “complete in view of the intended purpose” (Article 10(3)) “have the appropriate statistical properties, including, where applicable, as regards the persons or groups of persons in relation to whom the high-risk AI system is intended to be used” (Article 10(3)) “take into account, to the extent required by the intended purpose, the characteristics or elements that are particular to the specific geographical, contextual, behavioural or functional setting within which the high-risk AI system is intended to be used” (Article 10(4))^a^

^a^This requirement may be fulfilled by training and testing the system on “data reflecting the specific geographical, behavioral, contextual or functional setting within which they are intended to be used” (Article 42).

While the AIA’s aspirations are commendable, questions still arise about how these provisions are to be properly operationalized by medical AI systems providers in specific contexts. For example, as touched upon earlier, it may be unclear what population descriptors should be considered to ensure that the data are “sufficiently representative” of the intended population. Discussions and research on these issues are emerging^20^ with considerable efforts taken to address similar questions in genomic research.^21^ Soft law (ie, instruments that are not directly legally binding) such as guidelines and standards (see Chapter III, Section 5, AIA), are expected to prove important sources for understanding compliance in concrete scenarios. The standards will include technical solutions and details. Once harmonized (ie, their references are published in the Official Journal of the European Union), adherence to them may be used to achieve presumption of conformity with the relevant requirements of the AIA. Relevantly, the European Commission has issued standardization requests to the European Committee for Standardisation and European Committee for Electrotechnical Standardisation in support of the AIA.^22^ Among the requested standards are standards on “governance and quality of datasets used to build AI systems,” which should contain detailed requirements concerning these issues. Underscoring the difficulty of this work, standardization efforts within the AIA, the anticipated completion of requested standards has reportedly been pushed from August 2025 into 2026.^23^

## III. DEBIASING EXCEPTION

As mentioned above, limited availability of data, which is in part related to data protection laws, is also likely to present problems in ensuring high quality data. Article 10(5) aims to respond to this issue by introducing the so called “debiasing exception”. This exception essentially extends the conditions in which special categories of personal data (also referred to as “sensitive data”) including health data (see Table 4), which are afforded special protection under the General Data Protection Regulation (GDPR), can be lawfully processed. Specifically, as explained in Recital 70 of the AIA, the processing of sensitive data to ensure bias detection and correction falls within the scope of the GDPR’s “substantial public interest” exception allowing for processing sensitive data (Article 9(2)(g), GDPR) “to the extent that it is strictly necessary for the purpose of ensuring bias detection and correction,” provided that certain conditions are fulfilled. In other words, developers of AI systems may process sensitive personal data to detect and mitigate biases in datasets used to develop AI systems under the “substantial public interest” exception of the GDPR, if they meet the requirements of the GDPR and all six conditions outlined in Article 10(5) of the AIA.

**Table 4:** Special categories of personal data specified in Article 9(1) of the GDPR

**Special categories of personal data**
data revealing racial or ethnic origin political opinions religious or philosophical beliefs trade union membership genetic data biometric data (when processed for the purpose of uniquely identifying a natural person) data concerning health data concerning a natural person’s sex life or sexual orientation

These six conditions, however, are cumbersome and restrictive. The first condition states that sensitive personal data can only be used where “the bias detection and correction cannot be effectively fulfilled by processing other data, including synthetic or anonymized data.” Therefore, only when other avenues for debiasing have been exhausted or ruled out, and these avenues have been properly documented (Article 10(5)(f)), may sensitive personal data be processed for debiasing under this exception. The other conditions require the implementation of “technical limitations on the re-use of the personal data, and state-of-the-art security and privacy-preserving measures” (Article 10(5)(b)) and “measures to ensure that the personal data processed are secured, protected, subject to suitable safeguards” to prevent misuse and unauthorized use (Article 10(5)(c)). There are also conditions that prohibit accessing or transferring the data to third parties (Article 10(5)(d)), require their deletion as soon as bias correction is completed or the data’s retention period expires (Article 10(5)(e)), and justifying why the use of the data is strictly necessary in the data processing records (Article 10(5)(f)). As indicated by van Bekkum (2025), the Court of Justice of the European Union established a stringent standard for the “strict necessity,” which implies that, among other things, the providers should clearly define how the data will be used for bias detection and correction.^24^ These six conditions overlap to some degree with the GDPR’s provisions (eg, storage limitation principle). From the perspective of data subjects and patients, the conditions in Article 10(5) serve to ensure that their sensitive information is not frivolously re-purposed for providers’ debiasing efforts. The lawmakers have clearly taken efforts to minimize risks of a potential data breach.^25^ However, providers of medical AI systems may see the provision as overly restrictive. In particular, the condition of “strict necessity” may pose problems, given that currently there is no consensus on the routine use of sensitive data as attributes to ensure representativeness of a dataset, without prior indication of differences in performance between the groups. Notwithstanding, in addition to the “substantial public interest” exception, the GDPR provides other derogations for processing sensitive data (Article 9(2)), such as explicit consent of data subjects, which developers of AI systems may rely on when using sensitive data for debiasing purposes.

## IV. CONFORMITY ASSESSMENT, POST-MARKET REQUIREMENTS, AND ENFORCEMENT

Under the AIA, procedures and systems for data management must form part of the provider’s quality management system (ie, a set of policies, procedures, and instructions) (Article 17(1)(f), AIA), while a description of training, validation, and testing datasets must be included in the technical documentation (ie, documentation demonstrating compliance with the requirements for high-risk AI systems) (Annex IV, Section 2 (d) and (g), AIA). In the case of medical AI systems, the quality management system and technical documentation must meet the requirements of both the AIA and MDR/IVDR (Article 11(2) and 17(3), AIA). The quality management system and technical documentation will be assessed by a notified body (ie, a private entity tasked with conducting conformity assessments) in a conformity assessment procedure aiming to verify compliance with the requirements of the AIA and MDR/IVDR (Article 43(3), Annex VII, AIA). If the evaluation is successful, the provider can draw a declaration of conformity, start using the CE marking, and lawfully market and sell the medical AI system.

As part of the conformity assessment procedure (Annex VII, Section 4.3, AIA), the AIA grants notified bodies access to training, validation, and testing datasets (“[w]here relevant, and limited to what is necessary to fulfil its tasks”). This indeed may help to ensure that only medical AI systems built using data of appropriate quality enter the market. However, notified bodies may be hamstrung by data protection requirements in their access to the data used by the providers. In particular, training, validation, or testing data processed through the above-mentioned debiasing exception may be unavailable to the notified body to review due to the limitation of Article 10(5)(e), which demands that “the special categories of personal data are deleted once the bias has been corrected or the personal data has reached the end of its retention period, whichever comes first.” Consequently, the notified body may be unable to quality-check sensitive data used for debiasing, since the provider is obliged to promptly delete this data.

In the case of adaptive systems, data quality is relevant not only for the pre-market conformity assessment, but also once the AI system is in use. The AIA permits placing on the market adaptive systems, that is, systems “automatically adapting how functions are carried out” (Recital 128 AIA), provided a set of conditions is fulfilled. Amongst other things, providers must implement strategies ensuring that input data, which in this case simultaneously meet the AIA’s definition of training or validation data, used by the system to “learn” once in use meet the requirements for data quality (Annex IV, Section 2 (f); Article 15(4); AIA). To ensure, the post-market compliance providers of high-risk systems must implement a post-market monitoring system aiming to collect and analyze data on the performance of the AI system (Article 72, AIA).

While notified bodies are meant to ensure that only AI systems compliant with the AIA enter the market, national market surveillance authorities (MSAs) are formally the chief enforcers of the AIA. For medical AI systems, these will be the same MSAs as under the MDR/IVDR (Article 74(3), AIA). The MSAs’ function is to, among other things, ensure that products on the market provide “a high level of protection of public interests, such as health and safety” through activities such as checks on the characteristics of products (Articles 1(1) and 11(3), Regulation 2019/1020).^26^ The AIA bolsters MSA’s existing investigative and enforcement powers in the AI context. For example, they have the power to “be granted full access by providers to the documentation as well as the training, validation and testing data sets used for the development of high-risk AI systems” (Article 74(12), AIA). Similarly to the provisions concerning notified bodies discussed above, however, this prerogative raises concerns about the compatibility with the debiasing exception.

Beyond the traditional product safety powers granted to MSAs, the AIA also provides for MSAs and operators of AI systems to cooperate with fundamental rights agencies under certain circumstances, including anti-discrimination agencies likely to be relevant in the context of medical AI bias (Article 79(2), AIA). Additionally, under the AIA, anyone may submit a complaint about an infringement of the provisions of the AIA to a MSA (Article 85, AIA). This means that, for example, a patient or a physician may be able to report to a MSA an AI system which exhibits biased performance and raises concerns about the compliance with the data governance requirements. It is dubious, however, if that is a realistic scenario; consequently, in practice, the utility of the complaint mechanism for medical AI systems may be limited.

## V. CONCLUSIONS

Through its data governance provisions, the AIA directly addresses dataset bias, including its underlying causes. These requirements should help mitigate dataset bias in medical AI systems. Critically, the AIA does not prohibit the existence of biases in datasets or AI systems, recognizing that such a measure would be unrealistic. Rather, it focuses on ensuring that data management is “appropriate” given the intended use and context of the particular AI system.

The AIA’s data governance provisions are supported by its novel debiasing exception, and the data access powers granted to notified bodies and MSAs. These provisions go some way in responding to the challenge of data availability and the anticipated difficulties with enforcement. Both of these tools, however, are encumbered by limitations and further efforts may be needed to reconcile data protection concerns with the demands for data debiasing.

Data governance requirements seem a justified response to the potential negative impacts of dataset bias in medical AI systems. However, it should also be recognized that these provisions demand additional efforts from AI developers and notified bodies, which must also follow the comprehensive requirements of the MDR or IVDR. This will likely result in additional time and costs needed to introduce medical AI systems to the market.

The data quality provisions do not contain details, such as about the attributes (eg, age), which should be used to ensure representativeness of data, an omission which has seen criticism as a potential flaw of the AIA’s data quality provisions.^27^ However, given that the AI Act is horizontal, not sector-specific, and the debate on the use of appropriate attributes is ongoing, more specific provisions addressing these aspects could have been overly prescriptive and liable to redundancy. Associated soft law instruments, in particular the harmonized standard on “governance and quality of datasets used to build AI systems,” will likely address some of these details and practical aspects of the AIA’s data quality provisions. The issues that seem to merit particular attention in this context include the definition of “dataset bias,” population descriptors that should be used to ensure that data are sufficiently representative of the intended population; appropriate statistical properties of the data; and “characteristics or elements that are particular to the specific geographical, contextual, behavioural or functional setting” (Article 10(4)), which must be taken into account in the datasets. Data protection concerns, for example, the principle of data minimization embedded in the GDPR should be carefully considered in this context. As highlighted by the European Commission, the harmonized standards must be in conformity with the EU data protection law.^28^

Finally, although data bias is arguably the most important type of bias in medical AI systems, there are also other types and sources of bias to consider.^29^ Future research could focus, for example, on the implications and adequacy of the AIA’s provisions that address automation bias in a medical context.^30^

## Footnotes

1 Roxana Daneshjou et al., *Lack of Transparency and Potential Bias in Artificial Intelligence Data Sets and Algorithms: A Scoping Review*, 157 Jama  Dermatology, 1362 (2021).

2 Farhad Maleki et al., *Generalizability of Machine Learning Models: Quantitative Evaluation of Three Methodological Pitfalls*, 5 Radiology: Artificial  Intelligence e220028 (2023).

3 Derek L. Nguyen et al., *Patient Characteristics Impact Performance of AI Algorithm in Interpreting Negative Screening Digital Breast Tomosynthesis Studies*, 311 Radiology e232286 (2024).

4 Anurag Vaidya et al., *Demographic Bias in Misdiagnosis by Computational Pathology Models*, 30 Nature  Medicine 1174 (2024).

5 Beau Norgeot et al., *Minimum Information about Clinical Artificial Intelligence Modeling: The MI-CLAIM Checklist*, 26 Nature  Medicine 1320 (2020).

6 Xiaoxuan Liu et al., *Reporting Guidelines for Clinical Trial Reports for Interventions Involving Artificial Intelligence: The CONSORT-AI Extension*, 26 Nature  Medicine 1364 (2020).

7 Gary S. Collins et al., *TRIPOD + AI Statement: Updated Guidance for Reporting Clinical Prediction Models That Use Regression or Machine Learning Methods*, 385 BMJ e078378 (2024).

8 Gary S Collins et al., *Evaluation of Clinical Prediction Models (Part 1): From Development to External Validation*, 384 Bmj e074819 (2024).

9 Daniel Schwabe et al., *The METRIC-Framework for Assessing Data Quality for Trustworthy AI in Medicine: A Systematic Review*, 7 Npj  Digital  Medicine 203 (2024).

10 European Parliament and Council of the European Union, *Regulation (EU) 2017/745 of the European Parliament and of the Council of 5 April 2017 on medical devices, amending Directive 2001/83/EC, Regulation (EC) No 178/2002 and Regulation (EC) No 1223/2009 and repealing Council Directives 90/385/EEC and 93/42/EEC*, L 117 Official Journal of the European Union (2017). US Department of Health and Human Services, Health Insurance Portability and Accountability Act (1996).

11 European Parliament and Council of the European Union, *Regulation (EU) 2024/1689 of the European Parliament and of the Council of 13 June 2024 laying down harmonised rules on artificial intelligence and amending Regulations (EC) No 300/2008, (EU) No 167/2013, (EU) No 168/2013, (EU) 2018/858, (EU) 2018/1139 and (EU) 2019/2144 and Directives 2014/90/EU, (EU) 2016/797 and (EU) 2020/1828 (Artificial Intelligence Act)*, Official  Journal of the  European  Union (2024).

12 Hannah van Kolfschooten, *The AI Cycle of Health Inequity and Digital Ageism: Mitigating Biases Through the EU Regulatory Framework on Medical Devices*, 10 Journal of Law and the Biosciences (2023).

13 Marvin van Bekkum, *Using Sensitive Data to De-bias AI Systems: Article 10(5) of the EU AI Act*, 56 Computer Law & Security Review (2025).

14
Anu  Bradford, The  Brussels  Effect: How the  European  Union  Rules the  World (Oxford University Press. 2020).

15 Felix Busch et al., *Navigating the European Union Artificial Intelligence Act for Healthcare*, 7 Npj  Digital  Medicine 210 (2024). Emilia Niemiec et al., *Legal and Regulatory Challenges in Multi-Country Data-Driven Projects Developing and Validating Medical AI Systems in the EU*. 22 Expert  Review of  Medical  Devices, 9 (2025).

16 Emilia Niemiec, *Will the EU Medical Device Regulation help to improve the safety and Performance of Medical AI Devices?* 8 Digital Health DOI: 10.1177/20552076221089079 (2022). R. Beckers et al., *The EU Medical Device Regulation: Implications for Artificial Intelligence-Based Medical Device Software in Medical Physics*, 83 Physica  Medica 1 (2021). Niemiec, *supra* note 15.

17 Niemiec, *supra* note 15.

18 Busch, *supra* note 15.

19 David C. Higgins & Christian Johner, *Validation of Artificial Intelligence Containing Products Across the Regulated Healthcare Industries*, 57 Therapeutic  Innovation & Regulatory  Science 797 (2023).

20 Amelia Fiske et al., *Weighing the Benefits and Risks of Collecting Race and Ethnicity Data in Clinical Settings for Medical Artificial Intelligence,* 7 The Lancet Digital Health (2025).

21
National Academies of Sciences, Engineering, and Medicine, using Population Descriptors in Genetics and Genomics Research: A New Framework for an Evolving Field (2023).

22 European Commission, *Commission Implementing Decision of 22.5.2023 on a standardisation request to the European Committee for Standardisation and the European Committee for Electrotechnical Standardisation in support of Union policy on artificial intelligence C(2023)3215* (2023). European Commission, *Commission Implementing Decision of 23.6.2025 on a standardisation request to the European Committee for Standardisation and the European Committee for Electrotechnical Standardisation as regards high-risk AI systems in support of Regulation (EU) 2024/1689 of the European Parliament and of the Council and repealing Implementing Decision C(2023)3215* (2025)*.*

23 Euronews, *EU standards bodies flag delays to work on AI Act* (2025). Available at https://www.euronews.com/next/2025/04/16/eu-standards-bodies-flag-delays-to-work-on-ai-act.

24 Van Bekkum, *supra* note 13.

25 Marvin van Bekkum & Frederik Zuiderveen Borgesius, *Using Sensitive Data to Prevent Discrimination by Artificial Intelligence: Does the GDPR Need a New Exception?* 48 Computer Law & Security Review (2023).

26 The European Parliament and the Council of the European Union, *Regulation (EU) 2019/1020 of the European Parliament and of the Council of 20 June 2019 on market surveillance and compliance of products and amending Directive 2004/42/EC and Regulations (EC) No 765/2008 and (EU) No 305/2011 (Text with EEA relevance.)*, 169 Official  Journal of the  European  Union (2019).

27 van Kolfschooten, *supra* note 12.

28 European Commission, *supra* note 22.

29 Judy Wawira Gichoya et al., *AI Pitfalls and What Not to Do: Mitigating Bias in AI*, 96 British  Journal of Radiology (2023).

30 Johann Laux and Hannah Ruschemeier, *Automation Bias in the AI Act: On the Legal Implications of Attempting to De-Bias Human Oversight of AI* (January 30, 2025). Available at SSRN: https://ssrn.com/abstract=5117560 or http://dx.doi.org/10.2139/ssrn.5117560

